# Hyperaccumulation of Gadolinium by *Methylorubrum extorquens* AM1 Reveals Impacts of Lanthanides on Cellular Processes Beyond Methylotrophy

**DOI:** 10.3389/fmicb.2022.820327

**Published:** 2022-03-17

**Authors:** Nathan M. Good, Harvey D. Lee, Emily R. Hawker, Morgan Z. Su, Assaf A. Gilad, N. Cecilia Martinez-Gomez

**Affiliations:** ^1^Department of Plant and Microbial Biology, University of California, Berkeley, Berkeley, CA, United States; ^2^Department of Biomedical Engineering, Michigan State University, East Lansing, MI, United States; ^3^Division of Synthetic Biology, The Institute for Quantitative Health Sciences and Engineering, Michigan State University, East Lansing, MI, United States; ^4^Department of Microbiology and Molecular Genetics, Michigan State University, East Lansing, MI, United States; ^5^Department of Radiology, Michigan State University, East Lansing, MI, United States

**Keywords:** heavy metals, hyperaccumulator, methylotrophy, lanthanide, rare earth metals, biomineralization, RNAseq, MRI

## Abstract

Lanthanides (Ln) are a new group of life metals, and many questions remain regarding how they are acquired and used in biology. Methylotrophic bacteria can acquire, transport, biomineralize, and use Ln as part of a cofactor complex with pyrroloquinoline quinone (PQQ) in alcohol dehydrogenases. For most methylotrophic bacteria use is restricted to the light Ln, which range from lanthanum to samarium (atomic numbers 57–62). Understanding how the cell differentiates between light and heavy Ln, and the impacts of these metals on the metabolic network, will advance the field of Ln biochemistry and give insights into enzyme catalysis, stress homeostasis, and metal biomineralization and compartmentalization. We report robust methanol growth with the heavy Ln gadolinium by a genetic variant of the model methylotrophic bacterium *Methylorubrum extorquens* AM1, named *evo*-HLn, for “evolved for Heavy Lanthanides.” A non-synonymous single nucleotide polymorphism in a cytosolic hybrid histidine kinase/response regulator allowed for sweeping transcriptional alterations to heavy metal stress response, methanol oxidation, and central metabolism. Increased expression of genes for Ln acquisition and uptake, production of the Ln-chelating lanthanophore, PQQ biosynthesis, and phosphate transport and metabolism resulted in gadolinium hyperaccumulation of 36-fold with a trade-off for light Ln accumulation. Gadolinium was hyperaccumulated in an enlarged acidocalcisome-like compartment. This is the first evidence of a bacterial intracellular Ln-containing compartment that we name the “lanthasome.” Carotenoid and toblerol biosynthesis were also upregulated. Due to its unique capabilities, *evo*-HLn can be used to further magnetic resonance imaging (MRI) and bioremediation technologies. In this regard, we show that gadolinium hyperaccumulation was sufficient to produce MRI contrast in whole cells, and that *evo*-HLn was able to readily acquire the metal from the MRI contrast agent gadopentetic acid. Finally, hyperaccumulation of gadolinium, differential uptake of light and heavy Ln, increased PQQ levels, and phosphate transport provide new insights into strategies for Ln recovery.

## Introduction

The lanthanide series of elements (Ln) has recently been added to the life metals. A broader understanding of the functions of Ln in biology is slowly unfolding with discoveries of novel enzymes, metabolic pathways, and organisms that are dependent on these metals. Ln are known to form a cofactor complex with the prosthetic group pyrroloquinoline quinone (PQQ) for some alcohol dehydrogenase enzymes (Keltjens et al., 2014). To date, all known Ln-dependent metallo enzymes are from bacteria and coordinate the metal–PQQ complex for catalytic function. However, the physiological importance of PQQ stretches well beyond the prokaryotes. Mammals, including humans (Killgore et al., 1989), and plants (Choi et al., 2008) benefit from PQQ. Eukaryotes (Matsumura et al., 2014; Takeda et al., 2015) and archaea (Sakuraba et al., 2010) produce PQQ-dependent enzymes, though there is still much to be discovered regarding their activities and functions.

**FIGURE 1:**
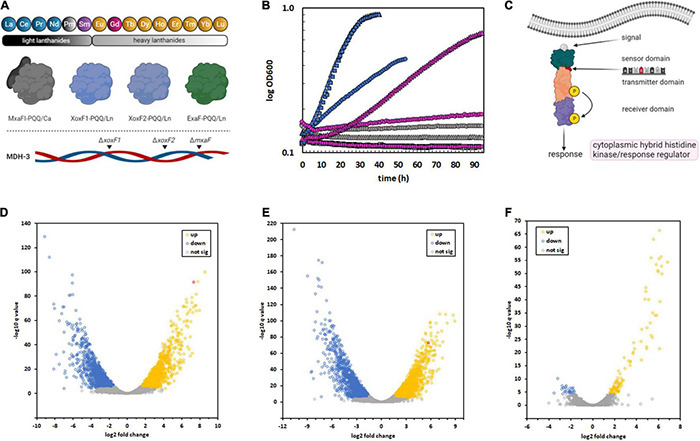
Isolation of an *M. extorquens* AM1 variant capable of metabolic utilization of Gd due to a SNP in a regulatory system, resulting in sweeping changes to gene expression. **(A)** *M. extorquens* AM1 is capable of methanol growth using the light Ln, excluding promethium (Pm), as part of the catalytic cofactor complex with PQQ. The genome encodes four distinct enzyme systems that can catalyze methanol oxidation, three of which are Ln-dependent. The MDH-3 mutant strain, which can only produce ExaF, was evolved for growth with Gd because of the flexibility of this enzyme (Good et al., 2016; Vu et al., 2016). **(B)** Growth of *M. extorquens* AM1 *evo*-HLn (triangles) with heavy Ln. Δ*mxaF* (squares), MDH-3 (circles). Strains were grown on methanol with either no Ln (gray), 2 μM Gd (pink), or 2 μM La (blue). Individual data points represent the mean of 9–12 biological replicates from 3 independent experiments. Variation among replicates for any given data point is within 5%. **(C)** A thymine-to-adenine transversion, effecting a leucine-to-histidine amino acid change in the sensory domain of a cytoplasmic hybrid histidine kinase/response regulator, was identified as the causative mutation allowing Gd growth by *evo*-HLn. This regulatory system was previously identified as being important for ExaF-dependent growth (Skovran et al., 2019). **(D–F)** The regulatory mutation in *evo*-HLn generates broad changes to gene expression that are both strain- and metal-dependent. Volcano plots show significantly altered expression patterns based on a stringent cutoff of log2 fold change of ± 1.5 and a false discovery rate adjusted *p*-value of < 0.0001. Comparison of the ancestral MDH-3 strain grown with La to *evo*-HLn reveals widespread transcriptional alterations with 2,415 DEGs with Gd **(D)** and 2,880 DEGs with La **(E)**. Gene expression changes between *evo*-HLn with Gd or La show a small subset of 137 DEGs specific to Ln species. When grown with La, *evo*-HLn **(F)**. Expression of *exaF* is highlighted in red in panels **(D,E)**.

XoxF methanol dehydrogenase (MDH) from the methylotrophic bacterium *Methylorubrum extorquens* AM1 was the first reported Ln-dependent metallo enzyme, and members of this diverse enzyme class are widespread in marine, fresh water, phyllosphere, and soil habitats (Nakagawa et al., 2012; Keltjens et al., 2014; Taubert et al., 2015; Chistoserdova, 2016; Huang et al., 2018; Ochsner et al., 2019). ExaF ethanol dehydrogenase was the first reported Ln-dependent enzyme with a preference for a multi-carbon substrate (Good et al., 2016), and its discovery has led to the identification of related enzymes in non-methylotrophic bacteria (Wehrmann et al., 2017; Wegner et al., 2019). Ln are also known to influence metabolic pathways in methylotrophic and non-methylotrophic bacteria (Good et al., 2019; Wehrmann et al., 2020), though studies to date have only assessed the impact of “light” Ln such as lanthanum (La; atomic number 57) or cerium (atomic number 58) (Figure 1A).

PQQ-MDH activity is critical for methylotrophic bacterial growth on compounds like methane and methanol (Dunstan and Anthony, 1972). Prior to the discovery of the role of Ln in methylotrophic metabolism, PQQ-MDHs were typified by MxaFI, an α_2_β_2_ tetrameric enzyme that coordinates calcium in the large subunit of each protomer (Adachi et al., 1990; Richardson and Anthony, 1992; Figure 1A). Light Ln are now known to play an important role in regulating MDH expression (Farhan Ul Haque et al., 2015; Chu and Lidstrom, 2016; Vu et al., 2016; Skovran et al., 2019), a phenomenon known as the “lanthanide-switch” in which the presence of light Ln downregulates expression of *mxa* genes and concomitantly upregulates expression of genes encoding Ln-dependent homodimeric dehydrogenase enzymes, such as XoxF and ExaF (Figure 1A). Exposure to light Ln generates modest alterations in gene expression, including downregulation of PQQ biosynthesis genes (Gu and Semrau, 2017; Masuda et al., 2018; Good et al., 2019), but has yielded limited insight into Ln chelation, transport, or granulation. Growth studies with mesophilic methylotrophs and the Ln series of metals have shown that only members of the “light” classification, ranging from La to neodymium (atomic number 60), can support growth with XoxF MDH similar to calcium with MxaFI MDH (Daumann, 2019). In comparison, methanol growth with samarium (atomic number 62) was much slower (Vu et al., 2016), and heavier Ln did not allow for growth. Intriguingly, the MDH-3 triple methanol dehydrogenase knockout mutant, retaining only the genetic capacity to produce ExaF to generate MDH activity (Figure 1A), grew comparatively better with samarium, raising the question of this enzyme’s compatibility with heavier Ln. In general, Ln of atomic numbers higher than samarium do not support methanol growth with a few exceptions (Daumann, 2019). For example, *Methylacidiphilum fumariolicum* SolV, which exhibits moderate growth with europium (atomic number 63) and limited growth with gadolinium (Gd; atomic number 64), grows optimally in acidic conditions (pH 2–5) where Ln are soluble, and as such does not have a known transport system for cytoplasmic uptake of these metals. In contrast, *M. extorquens* AM1 grows at neutral pH and has a TonB-dependent receptor (LutH), and a dedicated ABC transport system composed of a periplasmic protein (LutA), a membrane component (LutF), and an ATPase system (LutE) encoded in the *lut* (lanthanide-utilization and transport) gene cluster (Roszczenko-Jasińska et al., 2020). Genetic and phenotypic studies have shown that LutH is the primary transporter for light Ln into the periplasm, as it has been shown that a disruption of *mxaF* and *lutH* prevents growth with light Ln for about 100 h. The membrane and ATPase components from the ABC transporter, on the other hand, are essential for growth with light Ln and have been shown to facilitate Ln transport into the cytoplasm (Roszczenko-Jasińska et al., 2020). While growth rate on methanol correlates closely with MDH activity, it is still not known if this is due solely to enzyme catalysis or if transport of Ln ions is a limiting factor as well. If transport of heavy Ln is restricted, it is unknown how the cell differentiates between heavy and light Ln. Regardless of the factor(s) limiting growth, Gd seems to be the pivotal Ln marking the threshold of life with these metals (Daumann, 2019).

Gd is a versatile element that is widely used in industry (Ebrahimi and Barbieri, 2019) but is perhaps best known for its use as a contrast agent for magnetic resonance imaging (MRI). Its seven unpaired electrons give Gd unparalleled paramagnetic properties, making it the most effective agent for clinical application (Srivastava et al., 2015). Gd alone is highly toxic to humans (Le Fur and Caravan, 2019) and is therefore injected as a nine-coordinate ion chelated by an octadentate polyaminocarboxylate ligand with a water co-ligand (Wahsner et al., 2019). The stability of gadolinium-based contrast agents (GBCAs) makes them highly effective for intravenous delivery, and they are used in an estimated 30 million MRI exams annually (Lohrke et al., 2016), with approximately half a billion doses administered thus far (McDonald et al., 2018). While most GBCAs are excreted post-injection in urine, they are not innocuous. Over the past two decades, the development of nephrogenic systemic fibrosis has been observed in GBCA injection patients with impaired renal function, resulting in joint pain, immobility, and even death (Grobner, 2006; Marckmann et al., 2006; Boyd et al., 2007; High et al., 2007). In the last five years, concern has increased over the use of GBCAs with long-term retention found in patients with normal kidney function (Kanda et al., 2014, 2015; McDonald et al., 2015; Roberts et al., 2016). Anaphylactic shock and kidney failure have also been reported as possible outcomes of Gd accumulation in tissues (Ergün et al., 2006; Hasdenteufel et al., 2008). GBCAs are also cause for concern as rising anthropogenic Gd levels in surface water correlates with steadily increasing annual MRI exams worldwide (Ebrahimi and Barbieri, 2019). Due to the toxicity and rising concentrations of this micro contaminant, the potential health impacts on aquatic life and bioaccumulation in the food chain deserve more attention, as do wastewater treatment strategies that are sufficient to remove Gd.

In this study, we report a *M. extorquens* AM1 genetic variant that has gained the ability to grow robustly on methanol using the heavy Ln Gd. We identified a single non-synonymous substitution in a cytosolic hybrid histidine kinase/response regulator resulting in a gain-of-function mutation. The single-nucleotide polymorphism (SNP) was reconstructed in the ancestral background confirming that it was the causative mutation and fully regenerated the Gd-dependent growth phenotype. The variant exhibited broad alterations to its transcriptomic profile when grown with Gd or La, including upregulation of heavy metal stress response genes, central metabolism, and the biosynthetic gene clusters for PQQ, toblerol, carotenoids, and a proposed lanthanophore. Inductively coupled plasma mass spectrometry (ICP-MS) metal measurements show the evolved variant hyperaccumulated Gd in a trade-off for reduced accumulation of La, indicating mechanisms for biological differentiation of light and heavy Ln. Furthermore, Gd granules were contained in visible acidocalcisome-like “lanthasomes,” a structure that has not yet been reported. Together, these findings provide the first assessment of the impact of a heavy Ln on bacterial metabolism. Finally, we show evidence that the evolved variant can be used for the development of Gd bioremediation and Ln recovery applications by acquiring Gd from the GBCA gadopentetic acid, and for MRI technologies by generating contrast in vivo.

## Results

### Isolation of an *M. extorquens* AM1 Genetic Variant Capable of Gd-Dependent Methanol Growth

The MDH-3 mutant strain of *M. extorquens* AM1 can grow on methanol when provided an exogenous source of light Ln ranging from La to samarium. Knowing that the MDH-3 strain exhibited the best methanol growth with samarium (Vu et al., 2016), we chose to investigate the capacity of this strain for heavy Ln use as growth with Gd had not been tested prior to this study (Vu et al., 2016). Methanol minimal medium with Gd was inoculated and culture density was measured over time. No detectable increase in culture density was observed after 14 days of incubation at 30°C. However, after another 7 days of incubation, the MDH-3 culture density had increased ∼2.3-fold, reaching a final OD_600_ of 0.35 ± 0.03 (*N* = 4). Gd-grown cells were transferred to fresh methanol minimal medium with Gd and grown to maximum culture density. This process was repeated twice. Cells from these Gd-grown cultures were washed four times with sterile minimal medium to remove possible residual extracellular Gd, resuspended in 1 ml of sterile medium, and saved as freezer stocks with 5% dimethylsulfoxide at −80°C.

The long incubation time prior to growth of the original cultures with Gd suggested either a period of metabolic acclimation or genomic adaptation. To discern between these two possibilities, growth on methanol was tested after first passaging the strain three times on solid succinate medium and then inoculating into liquid succinate medium to generate pre-cultures. After growth, cells were harvested, washed four times with sterile minimal medium without a carbon source, and then inoculated into methanol medium with Gd. Growth was measured using a microplate spectrophotometer, and compared to the ancestral MDH-3 and Δ*mxaF* strains (Figure 1B). The Δ*mxaF* mutation ensures that the strain is dependent on Ln for methanol growth, and MDH-3 carries this mutation (Figure 1A). The variant strain exhibited growth using Gd within ∼10 h of inoculation, and a specific growth rate and maximum culture density like MDH-3 growth with La (Figure 1B). The lack of the two-week lag in growth that we observed after the ancestral MDH-3 inoculation with Gd was indicative that the underlying cause of growth with Gd was genomic adaptation rather than metabolic acclimation.

### Identification and Reconstruction of the Mutation Allowing Gd-Growth

Genomic DNA was isolated from the variant, sequenced, and analyzed for mutations. Three SNPs were identified in the variant (Supplementary Table 3), one of which was a non-synonymous thymine-to-adenine nucleotide transversion resulting in a leucine-to-histidine amino acid substitution in a hybrid histidine kinase/response regulator encoded at locus META1_1800 (Figure 1C). The gene META1_1800 was recently identified to affect ExaF function in *M. extorquens* AM1 in a transposon mutant hunt (Skovran et al., 2019). The mutation was confirmed by PCR amplification and Sanger sequencing analysis. The variant strain was named *evo*-HLn for “evolved for growth with heavy Ln.” Because META1_1800 integrity was previously shown to be necessary for ExaF function, we speculated that the T452A transversion in *evo*-HLn could be a gain-of-function mutation. The mutation was introduced into the MDH-3 genetic background by *sacB*-based allelic exchange. The reconstructed *evo*-HLn strain was named *evo*-HLn*^rec^*, and methanol growth dependent on Gd was confirmed (Supplementary Figure 1). Growth phenotypic analysis showed that the single-nucleotide substitution was able to fully reconstruct the Gd-dependent methanol growth rate and growth yield phenotypes observed for *evo*-HLn (Table 1).

**TABLE 1:** Growth rates and yields of strains grown in minimal medium with methanol with or without Ln.

Strain	Ln source^ε^	Growth rate^ϑ^	Growth yield^ϑ^
Wild type	None	0.15 ± 0.01	0.72 ± 0.13
Wild type	LaCl_3_	0.15 ± 0.01	0.78 ± 0.14
Wild type	GdCl_3_	0.14 ± 0.00	0.77 ± 0.14
MDH-3	None	n.d.	–
MDH-3	LaCl_3_	0.02 ± 0.00	0.41 ± 0.08
MDH-3	GdCl_3_	n.d.	–
Δ*mxaF*	None	n.d.	–
Δ*mxaF*	LaCl_3_	0.14 ± 0.01	0.90 ± 0.04
Δ*mxaF*	GdCl_3_	n.d.	–
*evo*-HLn	None	n.d.	–
*evo*-HLn	LaCl_3_	0.11 ± 0.01	0.93 ± 0.13
*evo*-HLn	GdCl_3_	0.03 ± 0.00	0.69 ± 0.04
*evo*-HLn*^rec^*	None	n.d.	–
*evo*-HLn*^rec^*	GdCl_3_	0.03 ± 0.00	0.63 ± 0.06

*^ε^Ln were provided in the growth medium at 2 μM.*

*^ϑ^Values represent the averages of 10 biological replicates from 3 independent experiments with standard deviations.*

*n.d., determined; –, no growth.*

*Culture density was monitored for up to 96 h.*

### Genetic Adaptation of *evo*-HLn Effects Broad Transcriptional Responses

To better understand the breadth of regulatory changes in *evo*-HLn, we analyzed transcriptomic profiles during methanol growth with Gd (*evo*-Gd) or La (*evo*-La) relative to MDH-3, the ancestral strain, grown with La (MDH3-La) and identified differentially expressed genes (DEGs) for each pairwise comparison. After adopting a stringent cutoff (greater than ± 1.5 log2 fold change in expression level, adjusted *p*-value < 0.0001), we identified 2,415 DEGs comparing *evo*-Gd to MDH3-La (Figure 1D) and 2,880 DEGs comparing *evo*-La to MDH3-La (Figure 1E). Among the highest upregulated genes in both comparisons was *exaF* (Figures 1D,E), indicating a likely increase in MDH activity corresponding with Gd growth. To identify genes that could be involved specifically in processes related to heavy Ln metabolism, *evo*-Gd was compared to *evo*-La and a smaller set of 137 DEGs was identified (Figure 1F). The large overlap of DEGs in *evo*-Gd and *evo*-La, when compared to MDH3-La, was indicative of the adapted hybrid histidine kinase/response regulator system being involved, directly or indirectly, in extensive transcriptional changes detected by RNA-seq. While this study was primarily focused on identifying changes to Ln metabolism, and in particular processes specific to heavy Ln, subsequent studies will characterize the two-component regulatory system in detail as this initial characterization suggests it regulates an expansive modulon.

### Increased *exaGJF* Expression Produces Higher MDH Activity

**FIGURE 2:**
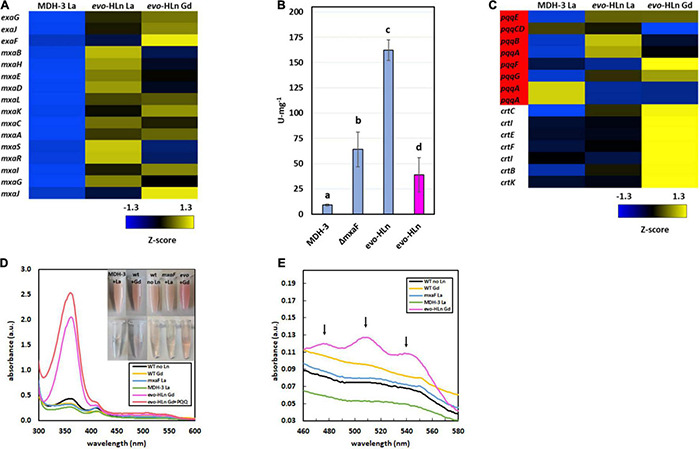
Gene expression patterns in *evo*-HLn provide insight into strategies for proliferation with Gd. **(A,C)** Heat maps show DEGs coded by Z-score of normalized read counts for key metabolic and physiological processes comparing methanol growth of *evo*-HLn with Gd or La to MDH-3 with La. **(A)** Expression of *exaGJF*, encoding ExaF alcohol dehydrogenase and accessory proteins, is increased in *evo*-HLn. In contrast, expression of *mxaF*, encoding the large subunit of the calcium-dependent MxaFI MDH, is switched off in *evo*-HLn, though several accessory genes, including the small subunit *mxaI*, are upregulated. **(B)** Methanol dehydrogenase activity is increased in *evo*-HLn, allowing for fast growth with La and robust growth with Gd. MDH activity was measured from cell extracts of MDH-3, Δ*mxaF*, and *evo*-HLn grown in methanol medium with either La (blue), or Gd (pink), and determined using the DCPIP dye-linked assay according to Anthony and Zatman with previously reported modifications (Anthony and Zatman, 1964; Vu et al., 2016). Bold letters indicate significance groups at *p* < 0.05 by one-way analysis of variance (ANOVA) and Tukey’s Honestly Significant Difference (HSD) test. **(C)** Expression of PQQ (red) is upregulated in *evo*-HLn, though Ln species differences indicate modifications to PQQ levels. Carotenoid biosynthetic genes are highly upregulated in *evo*-HLn in a Gd-specific manner. **(D)** The distinctive bright pink coloration of *evo*-HLn grown with Gd (top inset, cell suspensions; bottom inset, cell extract) is, in part, a result of increased PQQ levels. UV-Visible spectrum of cell extracts of wild type grown without Ln (black), wild type with 2 μM Gd (yellow), *evo*-HLn with 2 μM Gd (pink), and MDH-3 (green) and Δ*mxaF* (blue) with 2 μM La. Absorbance at 361 nm increases in the *evo*-HLn extract with the addition of 130 μM PQQ (red). Cell extracts were prepared in 25 mM Tris, pH 8.0. **(E)** Increased carotenoid levels also contribute to the color phenotype of *evo*-HLn. UV-Visible analysis shows absorbance peaks at 480 nm, 510 nm, and 540 nm (black arrows), which are characteristic of the carotenoid species synthesized by *M. extorquens* AM1 (Van Dien et al., 2003). These peaks are notably absent in all other conditions, including wild type cultured with Gd. For panels **(D,E)**, spectra represent the average of 3 separate replicates with extracts containing 5.3–5.6 mg/ml protein.

The *exaGJF* genes, encoding ExaF and accessory proteins, were highly upregulated with log2-transformed fold changes of 5.9, 4.6, and 7.3, respectively, for *evo*-Gd vs. MDH3-La. Surprisingly, the calcium-dependent MDH *mxa* accessory genes were also upregulated in *evo*-HLn (Figure 2A). This is distinct from a previous report for wild-type *M. extorquens* AM1 grown with La (Good et al., 2019) that is reliant on XoxF1 for methanol growth, suggesting additional regulation connecting ExaF and MxaFI oxidation systems. Since ExaF MDH activity is required for Ln-dependent methanol growth of MDH-3, one plausible explanation for the expanded range of metals used by *evo*-HLn was increased ExaF MDH activity, and this was supported by increased gene expression for this oxidation system. MDH activity was assayed in cell extracts of MDH-3, Δ*mxaF*, and *evo*-HLn prepared from cultures grown with methanol and either La or Gd. When grown with La, MDH activity in *evo*-HLn extracts was ∼18-fold higher than in MDH-3 extracts and ∼3-fold higher than extracts prepared from Δ*mxaF* (Figure 2B). All Ln species do not function equally well as part of the MDH cofactor complex, and the enzyme active site is finely tuned for light Ln (Jahn et al., 2018; Daumann, 2019). Though these studies were performed with XoxF MDH and the effects of different Ln species on ExaF activity are unknown, a reduction in ExaF MDH function with Gd in the active site was a reasonable expectation. Nonetheless, MDH activity was detectable in extracts of *evo*-HLn grown with Gd, corresponding to a ∼4-fold decrease compared to *evo*-HLn with La. Yet, this still constituted a fourfold increase in activity compared to MDH-3 with La (Figure 2B). It can be concluded then that increased ExaF MDH activity is a primary contributor to Gd-dependent methanol growth by *evo*-HLn. Through growth phenotypic analysis, it was found that wild type also grows on methanol with Gd (Supplementary Figure 2). Promoter fusion assays showed, however, that Gd did not induce *exaF* promoter activity in wild type (Supplementary Figure 3). Therefore, growth of wild type with Gd was reliant on calcium-dependent MxaFI, not ExaF, for MDH activity.

### Increased Pyrroloquinoline Quinone and Carotenoid Biosynthesis

Pyrroloquinoline quinone is essential for MDH activity in *M. extorquens* AM1, serving as part of the cofactor complex with a Ln or calcium ion. PQQ has also been shown to directly bind Ln (Lumpe and Daumann, 2019), suggesting a possible additional role as a lanthanophore. Expression of the PQQ biosynthesis genes was notably increased in *evo*-HLn, with some variation noted between the Gd and La conditions (Figure 2C). Cells of *evo*-HLn grown with Gd had a distinctive, bright pink coloration, and extracts prepared from *evo*-HLn cells retained this increased pigmentation (Figure 2D inset). When analyzed by UV-Visible spectrophotometry, *evo*-HLn extracts displayed a unique peak at 361 nm (Figure 2D). A peak around this wavelength is a signature of PQQ when bound to enzymes like ExaF (Good et al., 2016, 2019). To identify if PQQ contributed to the absorption anomaly, it was spiked into the *evo*-HLn extracts, which generated an observable increase in absorbance at the same wavelength. After normalizing for protein concentrations, the absorbance spectra indicated that PQQ in *evo*-HLn extracts was fourfold higher compared to wild type and sixfold higher compared to MDH-3 extracts. It could be expected that if PQQ does function as a secreted lanthanophore the concentration in the supernatant of *evo*-HLn would be higher. The absorbance at 360 nm in supernatants was ∼twofold higher in *evo*-HLn grown with Gd than MDH-3 with La.

It was also noted that carotenoid biosynthesis genes were uniformly highly upregulated in *evo*-Gd, suggesting that increased carotenoid production could be an underlying cause of the striking pigmentation. UV-Visible spectroscopy showed that *evo*-HLn grown with Gd had absorbance peaks at 480 nm, 510 nm, and 540 nm (Figure 2E) corresponding with the characteristic absorbance peaks of the carotenoid produced by *M. extorquens* AM1 (Van Dien et al., 2003). These peaks were absent in all other conditions tested, including wild type grown with Gd, showing that increased carotenoid production was dependent on *evo*-HLn and Gd.

### Multi-Pronged Transcriptional Response for Heavy Ln Uptake

**FIGURE 3:**
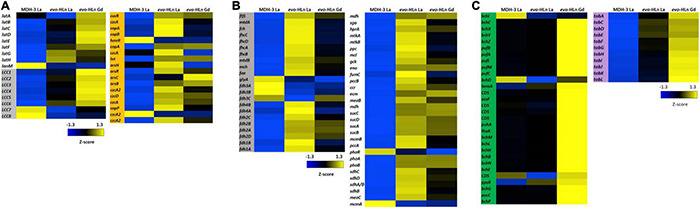
Upregulated gene clusters in *evo*-HLn for Ln acquisition, transport, and methylotrophy functions as well as bacteriochlorophyll and toblerol biosynthesis. **(A)** The presence of Ln increases expression of lanthanophore biosynthetic genes for acquisition (left, gray) and transport and utilization genes in *evo*-HLn (left, white), with Gd generating a stronger response. Ln also generate upregulation of heavy metal stress genes in *evo*-HLn (orange), though Gd induces a lesser response than La. **(B)** Methylotrophy genes for the conversion of formaldehyde, the oxidation product of methanol, to CO_2_ or the intermediate for assimilation (gray) and genes for assimilation into biomass (white) are upregulated. **(C)** Additional highly upregulated gene clusters in *evo*-HLn include bacteriochlorophyll biosynthetic genes (green), though their function is unknown, and genes for the biosynthesis of toblerol (purple).

Ln are transported to the periplasm via an outer membrane TonB-dependent receptor (LutH) and into the cytoplasm by a Ln-dedicated ABC transporter (LutA, LutE, and LutF). Both systems are encoded in the Ln utilization and transport (*lut*) gene cluster. The *lut* genes were upregulated in *evo*-HLn growing with Gd or La with the exceptions of *lutA*, encoding a periplasmic binding protein component of the ABC transporter, which was downregulated with La, and *lanM*, encoding a Ln/actinide-binding peptide, which was highly downregulated with either La or Gd (Figure 3A). Intriguingly, *lutH* was downregulated ∼2-fold in *evo*-Gd compared to *evo*-La, while *lutABCDEF* were all upregulated. This suggests that there are transcriptional modifications to reorganize the Ln transport machinery in *evo*-HLn with a stronger response for heavy Ln. Additionally, the gene cluster encoding a putative lanthanide chelator, known as the “lanthanide chelation cluster” (LCC), was upregulated in *evo*-HLn (Zytnick et al., 2022), with the exception of genes 7 and 8 (Figure 3A). As seen with the *lut* genes, expression levels of this lanthanophore-encoding cluster were highest in *evo*-Gd. Purification and characterization of the lanthanophore are currently underway. Upregulation of both a lanthanophore biosynthetic pathway and Ln transport machinery suggests that *evo*-HLn could be accumulating relatively higher levels of intracellular Ln.

In addition to upregulation of Ln acquisition machinery, *evo*-HLn exhibited a consorted transcriptional response to heavy metal stress. Heavy metal resistance genes were upregulated in *evo*-HLn, with a stronger response in the La condition than Gd (Figure 3A). Among these, *czc* genes, encoding a multi-metal cation-proton efflux pump, were among the most downregulated in *evo*-HLn with Gd compared to La, indicating that this resistance system may be dialed down, in turn allowing for Gd hyperaccumulation. These observations suggest that a modulated metal resistance response may contribute to cellular differentiation of light and heavy Ln.

### Upregulation of Methylotrophy and Biosynthetic Pathways

Primary oxidation genes, for the conversion of formaldehyde (the product of methanol oxidation by ExaF) for carbon assimilation, were on average upregulated 2.6-fold with Gd and 3.4-fold with La based on log2-transformed expression levels (Figure 3B). Only *glyA*, encoding serine hydroxymethyltransferase, and the genes encoding the periplasmic formate dehydrogenase *fdh3AB* were downregulated. Genes encoding the assimilatory serine cycle and ethylmalonyl-CoA pathways were also upregulated an average of 3.1-fold for Gd and 3.5-fold for La (Figure 3B). Only *phaR*, a gene encoding a polyhydroxybutyrate regulatory protein, and *mcmA*, encoding an assimilatory mutase, were downregulated. Expression of methylotrophy genes was increased in *evo*-La compared to *evo*-Gd, corresponding with faster growth (Figure 1B and Table 1).

Two groups of biosynthetic genes were among the most upregulated genes in *evo*-HLn. Genes for the biosynthesis of bacteriochlorophyll (Bchl), found in two separate clusters, were upregulated on average 5.7-fold for *evo*-Gd (Figure 3C). Mg^2+^ chelatase genes *bchI* and *bchD* were the only two genes in this group that were not upregulated and interestingly are involved in the insertion of Mg^2+^ into bacteriochlorophyll. The genome of *M. extorquens* AM1 does not appear to encode for a complete Bchl biosynthetic pathway as no bacteriochlorophyll synthase (*bchJ*) has been identified yet (Vuilleumier et al., 2009). The function of these genes, then, is currently unknown, though it is notable that they are only upregulated in *evo*-Gd. It can be speculated that the gene products may be involved in either energy production, based on the putative gene products, or remediation of oxidative stress due to the accumulation of the heavy Ln Gd. Higher carotenoid content in *evo*-HLn may be a stress response to genuine or perceived oxidative stress, and may be linked to the upregulation of bacteriochlorophyll biosynthetic genes like in plants (Ramel et al., 2012). Interestingly, *bch* genes and carotenoid genes were also upregulated with neodymium (Zytnick et al., 2022), but not with La (Good et al., 2019), indicating that heavier Ln have a greater influence on expression of these gene clusters.

The second most upregulated biosynthetic pathway is to produce the *Methylobacteria* antibiosis mediator toblerol (Ueoka et al., 2018). Biosynthetic genes *tobACDEFGHIJKL* were upregulated ∼8.3-fold for *evo*-Gd (Figure 3C), and were also upregulated to a lesser extent in *evo*-La compared to MDH3-La. It is possible that the hybrid histidine kinase/response regulator encoded by META1_1800 regulates, directly or indirectly, the *tob* biosynthetic cluster, as a stress response.

### Enhanced Ln Accumulation in *evo*-HLn

**FIGURE 4:**
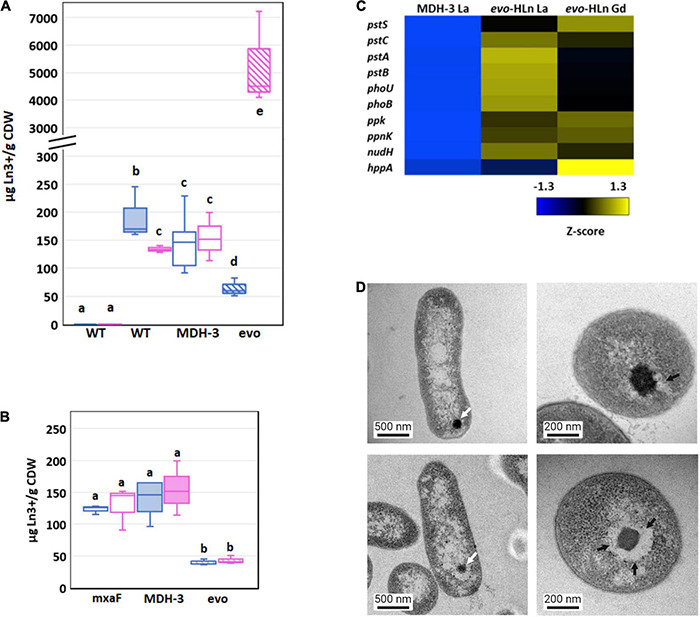
Genetic adaptation of *evo*-HLn generates a trade-off between light and heavy Ln accumulation **(A)** Comparison of light (La) and heavy (Gd) intracellular Ln content in wild type (shaded) and MDH-3 (open) strains shows only moderate differences, whereas *evo*-HLn (hatched) bioaccumulation exhibits a reduction in La and a striking increase in Gd. Cultures were grown on methanol minimal medium with 2 μM GdCl_3_, 2 μM LaCl_3_, or no lanthanides (Ln) as indicated. Gd (pink) and La (blue) contents are normalized to cell dry weight (CDW) for each growth condition. Box plots show interquartile range (boxes), median (line), and standard deviation (bars) for three biological replicates, each quantified three times by ICP-MS. **(B)** Availability of La impairs Gd uptake in *evo*-HLn, indicating additional machinery for heavy Ln acquisition and uptake. Intracellular Ln concentrations in the Δ*mxaF* mutant (open boxes), MDH-3 (shaded), and *evo*-HLn (hatched boxes) when cells were grown with both 2 μM GdCl_3_ and 2 μM LaCl_3_. Data were processed the same as in **(A)**. For panels **(A,B)**: wt, wild type; evo, *evo*-HLn; mxaF, Δ*mxaF*. **(C)** Polyphosphate genes are upregulated in *evo*-HLn indicating potential changes to polyphosphate metabolism and Ln-polyphosphate mineralization in this strain. Upregulated genes include the high-affinity phosphate transport system *pstSABC*, phosphate-specific transport system accessory protein encodin *phoU*, phosphate regulon regulatory gene *phoB*, and the H^+^-translocating pyrophosphate synthase gene *hppA*. **(D)** Lanthasomes are visible in *evo*-HLn as shown in TEM micrographs. White arrows show electron-dense Ln deposits (top left, Δ*mxaF*, La; bottom left, *evo*-HLn, Gd). Cross-sections (top right, Δ*mxaF*, La; bottom right, *evo*-HLn, Gd) show the surrounding lanthasome (black arrow) is larger and more defined in *evo*-HLn, indicating the increased capacity of Gd accumulation.

Increased ExaF MDH activity with upregulation of *lut* and LCC genes was suggestive of possible increased Ln accumulation in *evo*-HLn. Using ICP-MS, we quantified the Ln metal content of cells grown with methanol and a single Ln species. Gd was detected in wild type (Figure 4A), which was somewhat surprising. This shows that even heavy Ln are sensed, acquired, and stored by wild type even if they are not used catalytically or affect regulation of methanol oxidation systems. Uptake and storage by *evo*-HLn, on the other hand, was a striking ∼5 mg Gd/g cell dry weight constituting a 36-fold increase compared to wild type. The opposite trend, though much less pronounced, was observed when measuring accumulation of La with *evo*-HLn accumulating ∼3-fold less than the wild type. Comparing uptake and storage of each Ln within the same strain, we observed that *evo*-HLn accumulated ∼82-fold more Gd than La. The wild type, on the other hand, accumulated 0.4-fold more La than Gd. These data suggest that wild type prefers uptake and storage of light Ln, such as La, over heavy Ln. In contrast, increased Gd accumulation seen for *evo*-HLn indicates that it may have evolved a preference for heavy Ln. To test this possibility, we next compared Ln accumulation when the strains were provided equal concentrations of both La and Gd in the growth medium. Wild type accumulated equal amounts of La and Gd (Figure 4B). Total Ln content was ∼1.7-fold the amount of La and ∼2-fold the amount of Gd that was stored when only the single Ln species was provided (Figures 4A,B). Accumulation of each Ln species by *evo*-HLn was also similar when both were provided, but the overall levels were ∼3-fold less than what was observed for the wild type (Figure 4B). Total Ln content was ∼1.5-fold that of La alone for *evo*-HLn, but ∼63-fold less than the amount of Gd alone (Figures 4A,B). These data show that light Ln affects the capacity for *evo*-HLn to acquire and store heavy Ln and are suggestive of a second transport system for the latter.

Bioaccumulation of Ln by *M. extorquens* AM1 was reported previously by our group as electron-dense intracellular Ln deposits resembling polyphosphate granules (Roszczenko-Jasińska et al., 2020). Hyperaccumulation of Gd by *evo*-HLn could be generated by decoupling granulation from regulatory processes. Intriguingly, the *M. extorquens* AM1 genome does encode for an HppA P-type ATPase that is 73.2% identical to HppA from *Agrobacterium tumefaciens*, the identifying proton pump of bacterial acidocalcisomes (Docampo, 2006). Expression of *hppA* was upregulated 5-fold (log2-transformed expression) in *evo*-HLn with Gd (Figure 4C). Polyphosphate is important for Ln granulation and found in high concentrations in acidocalcisomes, though a recent study showed evidence for polyphosphate and acidocalcisomes being different structures (Frank and Jendrossek, 2020). Regardless, the polyphosphate genes in *evo*-HLn, including *ppk*, *ppnk*, *nudH*, and the high-affinity phosphate transporter genes *pstSABC*, were also upregulated (Figure 4C), providing corroborating evidence for Gd hyperaccumulation as polyphosphate deposits in acidocalcisome-like structures. Transmission electron microscopy (TEM) images of *evo*-HLn grown with Gd showed electron-dense granules within a vacuole (Figure 4D) that bears a striking resemblance to acidocalcisomes from *A. tumefaciens* (Seufferheld et al., 2003), which we call the “lanthasome.” Cross-cut TEM images show the uniformity and size of the lanthasome in *evo*-HLn, which are larger and more defined than those seen in Δ*mxaF* (Figure 4D).

### Gd Hyperaccumulation in *evo*-HLn Generates Contrast in Whole-Cell MRI Scans

**FIGURE 5:**
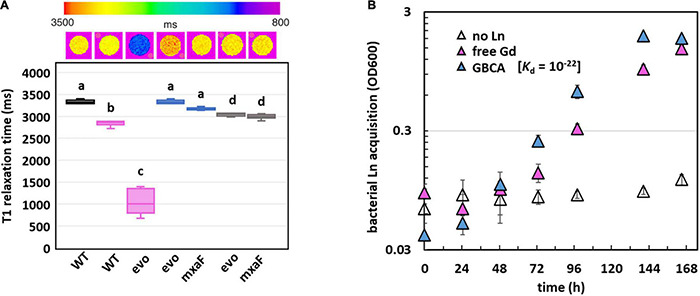
Gd hyperaccumulation by *evo*-HLn provides novel routes for potential MRI applications. **(A)** Gd hyperaccumulation generates significant contrast when scanned by MRI. Top shows digital scan with T1 relaxation times color coded. Bottom shows T1 relaxation times of cells grown without Ln (black), with La (blue), with Gd (pink), or with both (gray). For all plots, boxes represent the upper and lower quartiles of three independent biological replicate samples. Median lines and standard deviations (bars) are shown. Bold letters indicate different significance groups at *p* < 0.01 by one-way ANOVA and Tukey’s HSD test. **(B)** Gd is efficiently acquired from the strong chelating ligand DTPA by *evo*-HLn during methanol growth. Density of *evo*-HLn cultures is shown as triangles. Ln sources are denoted by color with various Ln sources: pink, 2 μM GdCl_3_; blue, 2 μM Gd-DTPA; open symbols, no Ln. Data points represent the average of 6 biological replicates from 2 independent experiments with error bars showing standard deviations.

Since Gd is a key component of GBCAs for MRI, we wondered if the quantity of Gd accumulated by *evo*-HLn could generate significant contrast. Gd-grown *evo*-HLn cells were washed to remove residual Gd ions and then scanned by MRI. Whole-cell scans generated a statistically significant reduction in T1 relaxation time for cells grown with Gd compared to cells grown with La or without Ln (Figure 5A). *evo*-HLn cells cultured with Gd displayed T1 that was > 3-fold less than wild type cultured without Ln. The T1 of wild-type cells with Gd was 0.2-fold less than cells without Ln. In contrast, intracellular accumulation of La by cells had a minor impact on T1 times (Figure 5A). When *evo*-HLn and Δ*mxaF* cells were grown with both Gd and La, T1 times were shortened by ∼0.1-fold compared to the no Ln treatment. These data show that MRI is sensitive enough to detect Gd accumulation in *M. extorquens* AM1 and that *evo*-HLn cells can accumulate Gd to intracellular concentrations that are high enough to produce robust MRI contrast.

### Efficient Acquisition of Gd From Gadopentetic Acid

Finally, the capacity of the *evo*-HLn strain to acquire Gd from the chelator diethylenetriamine pentaacetate (DTPA) was demonstrated (Figure 5B). Despite the high stability of the Gd-DTPA complex [log *K*_therm_ 22, log *K*_cond_ 17; (Tweedle et al., 1991; Wedeking et al., 1992)], *evo*-HLn was able to grow readily with no reduction growth rate compared to growth with soluble GdCl_3_ (Gd-DTPA, 0.04 h^–1^ ± 0.00; GdCl3, 0.03 h^–1^ ± 0.00; *n* = 3). This result indicates that *evo*-HLn has a highly effective means of sequestering Gd from DTPA, thus demonstrating a potential application in Gd recycling and remediation.

## Discussion

**FIGURE 6:**
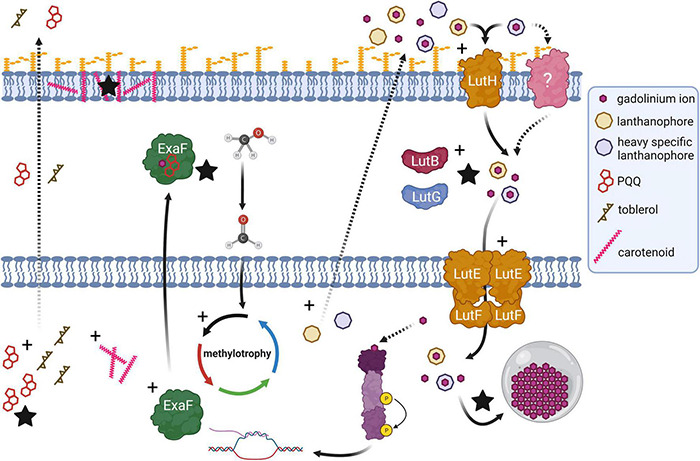
Genetic adaptation of a hybrid histidine kinase/response regulator (purple) in *evo*-HLn generates extensive transcriptional changes during methanol growth with the heavy Ln Gd, impacting methylotrophy, Ln metabolism, and biosynthetic pathways. Recognition of a signal molecule, possibly Ln and in this case Gd, by the regulatory system sensor domain triggers upregulation (black crosses) of methylotrophy pathways, including expression of *exaF* to generate higher MDH activity for the primary oxidation of methanol to formaldehyde. Biosynthetic pathways for PQQ and a Ln-chelating lanthanophore are also upregulated. More than one type of lanthanophore may be produced, and PQQ may play a role in Ln chelation. The Gd–lanthanophore complex is transported into the periplasm, by either the TonB-dependent receptor LutH or another receptor, or both. Gene expression profiling indicates disruption of the tight regulation of Ln uptake with upregulation of *lut* genes including those encoding the alternative periplasmic binding proteins LutF and LutG. Details of the state of Ln in the periplasm for transport to the cytoplasm, whether free or complexed with a lanthanophore, are not yet resolved. Increased Ln acquisition and trafficking leads to higher levels of Ln granulation in the cytoplasmic lanthasome (gray circle). Biosynthesis genes for carotenoids and toblerol are also upregulated. Higher carotenoid content contributes to the striking bright pink coloration of *evo*-HLn cells with Gd, and likely serves as an antioxidant response to heavy metal exposure. Production of toblerol may also be a stress response, as this cyclopropanol-containing molecule modulates antibiosis in *Methylobacteria*. Dashed arrow represents a proposed/unverified function. Black stars indicate experimentally validated increased functions.

This study has revealed much regarding evolutionary strategies for life with heavy Ln and their implications for Ln-biochemistry. Variant *evo*-HLn is adapted for growth using heavy Ln, a physiological feat that was not formerly possible by *M. extorquens* AM1. Though wild type *M. extorquens* AM1 can transport and accumulate Gd, growth is mediated by calcium-dependent MxaFI. *evo*-HLn transports and accumulates Gd to a much greater extent and exhibits wide-ranging transcriptional and physiological responses as a result. Differential gene expression analysis and validation allow for the generation of a model for *evo*-HLn with Gd whereby the metal is brought into the cell via one or more lanthanophore(s) (specific or promiscuous), is transported to the cytoplasm, and effects expansive transcriptional changes to the cell, possibly by acting as the signal for the regulatory system encoded by META1_1800 (Figure 6). Upregulation of metal resistance genes, carotenoid biosynthesis, bacteriochlorophyll biosynthesis, and toblerol biosynthesis are indicative of a broad stress response.

We identified that the gene cluster for one lanthanophore, the LCC, is upregulated. Yet, it may not be the only one. Levels of PQQ, a molecule already shown to bind Ln in solution (Lumpe and Daumann, 2019), are increased in *evo*-HLn, indicating a possible role for binding Ln and/or serving as a signal for their uptake. This is more striking given that PQQ biosynthesis genes are downregulated in wild type grown with La (Good et al., 2019). Increased Ln transport by *evo*-HLn suggests disruption of the tightly controlled regulatory mechanisms governing uptake (Roszczenko-Jasińska et al., 2020), and this is supported by differential expression of specific *lut* genes. *M. extorquens* AM1 produces multiple Ln-binding proteins for uptake and utilization encoded in the *lut* gene cluster. It is likely that *evo*-HLn uses some or all of this machinery to grow with Gd, such as alternative periplasmic binding proteins LutB and LutG. The Ln accumulation data reported in this study, however, also suggest that there may be alternative or additional machinery for uptake of heavy Ln that could include novel lanthanophores and novel lanthanophore receptors. Notably, the genes encoding the Pst phosphate transport system were highly upregulated in *evo*-HLn, and uptake of heavy Ln, such as Gd, may be tied to phosphate transport.

Increased Gd uptake by *evo*-HLn results in a reduced capacity to incorporate La, and if an alternative uptake system is predominant, this will allow us to define selectivity among heavy and light Ln. Mutational analysis of the *lut* genes has already provided evidence of alternative transport systems for light Ln (Roszczenko-Jasińska et al., 2020). Wild type and MDH-3 can transport and accumulate Gd intracellularly, but it is not sufficient to function as a signal for the switch from MxaFI MDH to a Ln-dependent oxidation system. We were not yet able to pinpoint a heavy Ln transport system in this study; however, these observations are suggestive of additional components, including regulatory elements, being co-opted for Gd acquisition, uptake, transport, and/or use in *evo*-HLn. The details of the molecular processes and regulation of storage of Ln by *M. extorquens* AM1 are still unknown, but Ln hyperaccumulation by *evo*-HLn provides an excellent comparator to the wild-type and ancestral strains to investigate these questions in depth.

The identification of lanthasomes for intracellular Ln storage provides an exciting insight into Ln storage. Lanthasomes in *evo*-HLn appear to be larger, with a more uniform and defined structure than those from Δ*mxaF*, and therefore this strain will serve as an excellent model for their study. Mechanistic details for lanthasome formation, structure, and function remain to be resolved, and work is ongoing to investigate these questions. The physiological reason underlying Ln storage is not yet known and being able to manipulate lanthasome formation will facilitate studies addressing their function.

It is common practice to inject patients with GBCAs before MRI to enhance scan effectiveness by shortening the T1 relaxation of the target tissue. Moreover, Gd is conjugated to proteins for applications such as blood pool agents, which can be done chemically (Caravan, 2006) *via* avidin-biotin (Dafni et al., 2002; Gilad et al., 2005) and genetically, *via* Ln binding tags (Xue et al., 2015). Alternatively, GBCAs are used as substrates for genetically encoded reporters (Nyström et al., 2019). The genetic variant *evo*-HLn has acquired the ability to transport and accumulate Gd such that a significant reduction in T1 relaxation can be observed by MRI in vivo. Magneto-endosymbionts, iron accumulating bacteria, are currently being developed as MRI contrast agents (Vargas et al., 2018). The discovery that *evo*-HLn can be used as an agent with superior contrast due to the unique paramagnetic properties of Gd opens new avenues for the development of bio-based contrast agents.

Although Gd is considered safe when properly chelated, deposition of unmetabolized GBCAs correlates with detrimental health effects in humans. We showed that *evo*-HLn grows readily on methanol with Gd-DTPA as the sole Ln source, revealing that *M. extorquens* AM1 must produce molecular machinery, likely including secreted lanthanophore(s), capable of sequestering Ln from highly stable complexes. Multiple lines of evidence show that *evo*-HLn transports and stores more Ln than the ancestral strain, indicating alterations to sequestration, uptake, and/or storage machinery. We are currently investigating candidate genes involved in each of these processes, including for the formation of lanthasomes. Understanding the mechanisms behind these individual processes will aid in future efforts to engineer efficient Ln-binding agents. Finally, the capacity for growth with GBCAs, increased uptake, and hyperaccumulation of Gd make *evo*-HLn an excellent candidate for the development of a microbial platform for recovery of Gd from wastewater and possibly even medical waste, a yet untapped reservoir for recycling of this critical metal.

## Materials and Methods

### Strains and Culture Conditions

*Methylorubrum extorquens* AM1 strains were routinely grown at 30°C in MP minimal medium (Delaney et al., 2013) with 15 mM succinate, shaking at 200 rpm on an Innova 2300 platform shaker (Eppendorf, Hamburg, Germany). For growth studies, 50 mM methanol was used as the sole carbon and energy source. Ln were added as chloride salts or gadopentetic acid (Gd-DTPA; Magnevist^®^) to a working concentration of 2 or 20 μM as indicated. When necessary, 50 μg/ml kanamycin was added to the growth medium for plasmid maintenance. Strains and plasmids used in this study are listed in Supplementary Table 1.

### Plasmid Construction

The META1_1800 gene sequence from *evo*-HLn, with an additional 400 bp of upstream sequence, was amplified from gDNA by PCR and cloned into pCM433KanT that had been linearized by PCR. Primers for both the insert and backbone fragments were designed to have complementary 20-bp overlaps (Supplementary Table 2). The insert and backbone fragments were assembled in electrocompetent *Escherichia coli* (Jacobus and Gross, 2015). Kanamycin-resistant clones were screened by colony PCR to confirm successful assembly. Confirmed assemblies were sequenced and verified, and the plasmid was named pNG341.

### Strain Construction

*M. extorquens* AM1 strains were transformed by electroporation (Toyama et al., 2006). After 24 h of outgrowth, transformants were selected by plating on MP medium with 1.5% agar, 15 mM succinate, and 50 μg/ml kanamycin or 20 μg/ml tetracycline. Transformants were incubated at 30°C until isolated colonies appeared. In–out allelic exchange to generate the *evo*-HLn mutation was performed as described using 5% sucrose for counterselection (Marx, 2008). After sucrose counterselection, successful reconstruction of the *evo*-HLn causative mutation was verified by PCR amplification and Sanger sequencing of the entire META1_1800 locus.

### Methanol Growth Analysis With Light and Heavy Lanthanides

*M. extorquens* AM1 strains were grown with succinate overnight, pelleted by centrifugation at 1,000 × *g* for 10 min at room temperature using a Sorvall Legend X1R centrifuge (Thermo Scientific, Waltham, MA, United States), and washed in 1 ml of sterile MP medium with methanol. For growth analysis in microplates, washed cells were resuspended in 200 μl of MP methanol medium and 10 μl was transferred to each microplate well with 640 μl of MP methanol medium. For growth studies with Gd-DTPA, 50 μl of inoculum was added to 3 ml of MP methanol medium in sterile 14-ml polypropylene culture tubes (Fisher Scientific, Hampton, NH, United States). Culture densities were monitored over time by measuring light scatter at 600 nm using either a Synergy HTX multi-mode plate reader (Biotek, Winooski, VT, United States) or an Ultraspec 10 density meter (Biochom, Holliston, MA, United States).

### UV-Visible Spectrophotometry

To prepare cell-free extracts, 50 ml of methanol-grown culture with Gd or La were harvested, upon reaching an OD_600_ of ∼1.1–1.3, by centrifugation at 4,696 × *g* for 10 min at 4°C. The supernatant was removed, and cell pellets were resuspended in 1.5 ml of 25 mM Tris, pH 8.0, and lysed using an OS Cell Disrupter at 25,000 psi (Constant Systems Limited, Low March, Daventry, Northants, United Kingdom). Lysates were transferred to 1.5-ml Eppendorf tubes and clarified of cell debris by centrifugation at 21,000 × *g* for 10 min at 4°C. Cell-free extracts were transferred to new 1.5-ml microcentrifuge tubes and kept on ice until needed. PQQ was prepared fresh to a working concentration of 5.3 mM in an opaque conical tube and kept on ice until needed. Absorbance spectra were measured from 250 to 600 nm with a Synergy HTX multi-mode plate reader. A blank buffer spectrum was subtracted as background. Protein concentrations were determined by absorbance at 280 nm and the bicinchoninic acid assay (Thermo Fisher Scientific, Waltham, MA, United States).

### Genomic DNA Extraction and Sequencing

*evo*-HLn was grown in shake flasks with 50 ml of MP with succinate to OD_600_ ∼ 1.0. Genomic DNA was extracted according to the “Bacterial genomic DNA isolation using CTAB” protocol (Joint Genome Institute, Walnut Creek, CA, United States). Samples were submitted to Genewiz (South Plainfield, NJ, United States) for whole genome sequencing using the Illumina HiSeq platform with 2 × 150 bp read length. Variant calling and analysis were performed by Genewiz using wild-type *M. extorquens* AM1 (genome assembly GCA_000022685.1) as the reference and Δ*mxaF* [the genetic background used to generate MDH-3 (Good et al., 2016)] as an additional comparator.

### RNA-Seq Transcriptomics

RNA samples were prepared as described previously (Good et al., 2019). After RNA extraction, samples were processed by the Microbial Genome Sequencing Center (Pittsburgh, PA, United States) for Illumina stranded library preparation, RiboZero Plus rRNA depletion, and 2 × 50 bp paired-end RNA sequencing. Raw sequencing data were processed on KBase (Arkin et al., 2018) using HiSAT2 for read alignment, StringTie for alignment assemblies, and DESeq2 for differential expression analysis.

### Methanol Dehydrogenase Activity Assays

Cell extracts were prepared as described above, but with an additional wash step in 20 ml of 100 mM Tris–HCl, pH 9.0, before disruption. Protein concentrations of cell-free extracts were determined by BCA assay. Methanol dehydrogenase activity was measured by monitoring the phenazine methosulfate (PMS)-mediated reduction of 2,6-dichlorophenol indophenol [DCPIP; ε_600nm_ = 21 mM^–1^ cm^–1^ (Good et al., 2016, 2019, 2020)] as described (Anthony and Zatman, 1967; Ghosh and Quayle, 1979; Vu et al., 2016; Good et al., 2020). To reduce background activity, all assay reagents were dissolved in water; PES and DCPIP solutions were prepared in opaque tubes and kept on ice; and cell-free extracts were pre-incubated for 2 min at 30°C as recommended (Jahn et al., 2020).

### Whole-Cell MRI

Wild-type and *evo*-HLn mutant strains were grown in 50 ml of methanol medium to maximal culture density (OD_600_ ∼3). No Ln was added to the medium for the wild-type strain. Either Gd, La, or both (2 μM each) were added to the medium for *evo*-HLn. Cells were harvested by centrifugation at 4,696 × *g* for 10 min at room temperature. The supernatant was removed, cells were washed two times by resuspension with 50 ml of 25 mM Tris, pH 7.0, and centrifugation at 4,696 × *g* for 10 min at room temperature. After washing, 1/10 of the cell pellets were resuspended in 500 μl of 25 mM Tris, pH 7.0. MRI data were acquired using a 7.0-T horizontal MRI (Bruker) equipped with a multi-channel receive array and volumetric transmit coil ensemble. T1 maps were generated via Paravision 360, with 10 TRs ranging from 400 to 17,500, TE = 6.89, MTX = 128/128, FOV = 32 cm^2^, ST = 1 mm and AVG = 3.

### Transmission Electron Microscopy

Samples were fixed in 2% glutaraldehyde in 0.1 M sodium cacodylate buffer, pH 7.2 (EMS, Hatfield, PA, United States), for at least 1–2 h at room temperature with agitation. Samples were rinsed [3×; 10 min, room temperature (RT)] in 0.1 M sodium cacodylate buffer, pH 7.2, and immersed in 1% osmium tetroxide with 1.6% potassium ferricyanide in 0.1 M sodium cacodylate buffer for 2 h. Samples were rinsed (3×; 10 min, RT) in buffer and then briefly in distilled water (1×; 1 min, RT). Samples were then subjected to an ascending acetone gradient (10 min; 35%, 50%, 70%, 80%, and 90%) followed by pure acetone (2×; 10 min, RT). Samples were progressively infiltrated with Epon resin (EMS, Hatfield, PA, United States), while rocking, and then polymerized at 60°C for 24–48 h in silicon molds. Thick sections (200 nm) were cut using a Reichert Ultracut E (Leica, Wetzlar, Germany), collected on glass slides, stained with toluidine blue, and used for general orientation. Thin sections (70 nm) were collected onto formvar-coated 50-mesh copper grids or slot grids. The grids were post-stained with 2% uranyl acetate followed by Reynold’s lead citrate, for 5 min each. The sections were imaged using a Tecnai 12 120-kV TEM (FEI, Hillsboro, OR, United States) and data were recorded using an UltraScan 1000 with Digital Micrograph 3 software (Gatan Inc., Pleasanton, CA, United States).

### Intracellular Ln Quantification

After whole-cell MRI analysis, cell pellets were dehydrated at 65°C for 72 h. Dried pellets were weighed before deconstruction in *Aqua regia* diluted in 2% nitric acid and sonicated for 0.5 h before passing through 0.45-μm Whatman syringe filters. Metal contents were determined by ICP-MS at the Laboratory for Environmental Analysis (Center of Applied Isotope Studies, University of Georgia).

## Data Availability Statement

The datasets presented in this study can be found in online repositories. The names of the repository/repositories and accession number(s) can be found below: National Center for Biotechnology Information (NCBI) BioProject database under accession number GSE193171.

## Author Contributions

NG, NM-G, and AG: conceptualization. NG: methodology. NG, HL, EH, and MS: investigation. NG and HL: writing—original draft. NG, NM-G, AG, and HL: writing—reviewing and editing. NM-G and AG: funding acquisition and resources. All authors contributed to the article and approved the submitted version.

## Author Disclaimer

The views and opinions of authors expressed herein do not necessarily state or reflect those of the United States Government or any agency thereof.

## Conflict of Interest

NM-G and NG are inventors on patent application B21-146 submitted by the Regents of the University of California. The remaining authors declare that the research was conducted in the absence of any commercial or financial relationships that could be construed as a potential conflict of interest.

## Publisher’s Note

All claims expressed in this article are solely those of the authors and do not necessarily represent those of their affiliated organizations, or those of the publisher, the editors and the reviewers. Any product that may be evaluated in this article, or claim that may be made by its manufacturer, is not guaranteed or endorsed by the publisher.
