# Supplementary material for: Hyperaccumulation of Gadolinium by Methylorubrum extorquens AM1 Reveals Impacts of Lanthanides on Cellular Processes Beyond Methylotrophy
Source: Front Microbiol. 2022 Mar 17;13:820327. doi: 10.3389/fmicb.2022.820327 (PMC8969499; doi:10.3389/fmicb.2022.820327)
Supplement: Supplementary file 2 [file Data_Sheet_2.PDF]

Supplementary Material

1 Supplementary Methods

1.1 Transcriptional reporter fusion assays

Strains carrying VENUS *yfp* fusion constructs were grown on methanol in 48-well microplate format. Upon reaching a culture density of OD_{600} ~0.35, 200 μL of culture were transferred to an optical bottom black 96-well plate. Fluorescence was measured at an excitation wavelength of 485 nm and an emission wavelength of 520 nm. Relative fluorescence units (RFU) were calculated as raw fluorescence ($F_{520\ nm}$) divided by OD_{600}.
Reporter-fusion assays previously showed that *exa* promoter activity was stimulated by La (Skovran, Raghuraman, and Martinez-Gomez 2019). Using the same promoter-reporter system, we replicated *exa* promoter stimulation with La in MDH-3 grown on methanol (~120 RFU), and repression of promoter activity in wild type and Δ*mxaF*. The *exa* promoter was assayed for activity in *evo*-HLn grown on methanol with La and observed a 275-fold increase compared to MDH-3. Promoter activity with Gd from *evo*-HLn increased a striking 470-fold compared to MDH-3 with La. Further, we did not detect *exa* promoter activity in wild type with Gd, showing that although wild type grows with methanol in the presence of Gd (Fig. S2), the switch from MxaFI to ExaF oxidation systems does not occur. This could be indicative of either wild type being unable to transport Gd or Gd not functioning as a signal for the “Ln switch” in this strain. Regardless, it can be concluded that wild type grows on methanol using MxaFI MDH, the Ca/PQQ-dependent oxidation system, when Gd is present in the medium.

2 Supplementary Data

2.1 Supplementary Tables

Table S1. Bacterial strains and plasmids used in this study

strain or plasmid	description	reference
**strains**		
Methylorubrum extorquens		
AM1	wild type; rifamycin-resistant derivative	(Nunn and Lidstrom 1986)
MDH-3	Δ*mxaF* Δ*xoxF1* Δ*xoxF2* triple deletion mutant	(Vu et al. 2016)
Δ*mxaF*	deletion mutant	(Marx et al. 2003)
evo-HLn	MDH-3 genetic variant adapted for methanol growth with heavy lanthanides	this study
evo-HLn^{rec}	reconstructed *evo*-HLn strain	this study

**plasmids**		
pCM433KanT	*sacB*-based allelic exchange vector, Km^r	(Puri et al. 2015)
pNG341	pCM433KanT with *evo*-HLn META1_1800 allele	this study
pAP5	promoterless *yfp* fusion vector, Tc^r	(Skovran et al. 2011)
pHV3	pAP5 with *exa* promoter region, Tc^r	(Skovran, Raghuraman, and Martinez-Gomez 2019)

Table S2. Primers used for plasmid construction

primer name	sequence
pCM433KanT For	CGAACTCGTCCACAAGCCGAATGTGCAGGTTGTCGGTGTC
pCM433KanT Rev	CGGCTGATCGAGCGGCAGTATGGTAACTGTCAGACCAAGT
META1_1800 For	ACTTGGTCTGACAGTTACCATACTGCCGCTCGATCAGCCG
META1_1800 Rev	GACACCGACAACCTGCACATTCGGCTTGTGGACGAGTTCG

Table S3. Mutations detected by genome resequencing of *evo*-HLn and Δ*mxaF*

Wild type *M. extorquens* AM1 genome assembly ASM2268v1 was used as the reference strain for mapping. Yellow highlights indicate single nucleotide variant and deletion mutations unique to *evo*-HLn. All other mutations shown were identified in both *evo*-HLn and Δ*mxaF*.

Chromosome	Region	Type	Ref	Allele	Count	Freq	Qual	locus_tag	Coding change	Amino acid change	Non
CP001510	482893^482894	In	-	C	47	100	200	META1p0458			-
CP001510	1673173	SNV	A	G	110	100	200	META1p1592	69T>C		No
CP001510	1873778	SNV	T	A	175	100	200	META1p1800	452T>A	Leu151His	Yes
CP001510	2329711	Del	G	-	125	98	160				-
CP001510	2777457	SNV	T	G	38	97	160	META1p2648	408A>C		No
CP001510	2803789	SNV	C	T	32	100	200	META1p2676	.63C>T		No

CP001510	2803840	SNV	T	C	9	100	155	META1p2676	114T>C			No
CP001510	2891642	SNV	G	C	185	100	200	META1p2763	879C>G			No
CP001510	3037769	Del	C	-	190	95	200	META1p2908	718delC		Arg241fs	Yes
CP001510	3159071	Del	G	-	94	97	160					-
CP001510	4001527.. 4001531	Del	CGT GC	-	122	85	200	META1p3891, META1p3892	262_266delG CACG		Ala88fs	Yes
CP001511	580985^5 80986	In	-	C	128	98	200	META2p0619	468_469insG		Arg157fs	Yes
CP001511	770863	Del	G	-	52	95	160	META2p0816	890delG		Ala298fs	Yes

2.2 Supplementary Figures

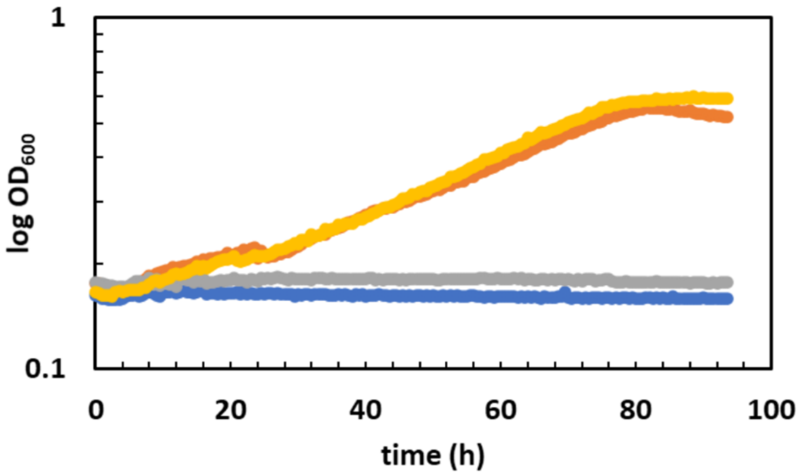

Supplementary Figure 1. Reconstruction of Gd^{3+}-dependent methanol growth. *evo*-HLn was inoculated into minimal methanol medium with (orange) or without 2 µM Gd^{3+} (blue). The reconstructed mutant strain, *evo*-HLn^{rec}, was tested for growth in the same conditions: with 2 µM Gd^{3+} (yellow), without Gd^{3+} (gray). Data points are the mean of 18 biological replicates from 2 independent experiments. Individual measurements for each data point are within 5% of one another.

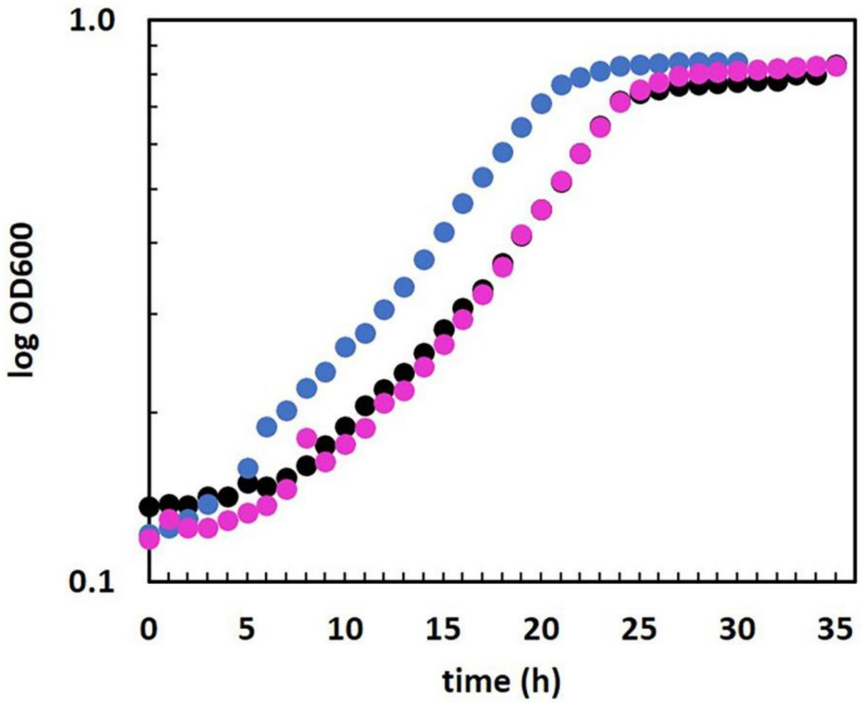

Supplementary Figure 2. Methanol growth of wild type *M. extorquens* AM1 with and without Ln^{3+}. Growth with no Ln^{3+} (black), 2 μM Gd^{3+} (pink) or 2 μM La^{3+} (blue). Data points are the mean of 10 biological replicates from at least 3 independent experiments. Individual measurements for each data point are within 5% of one another.

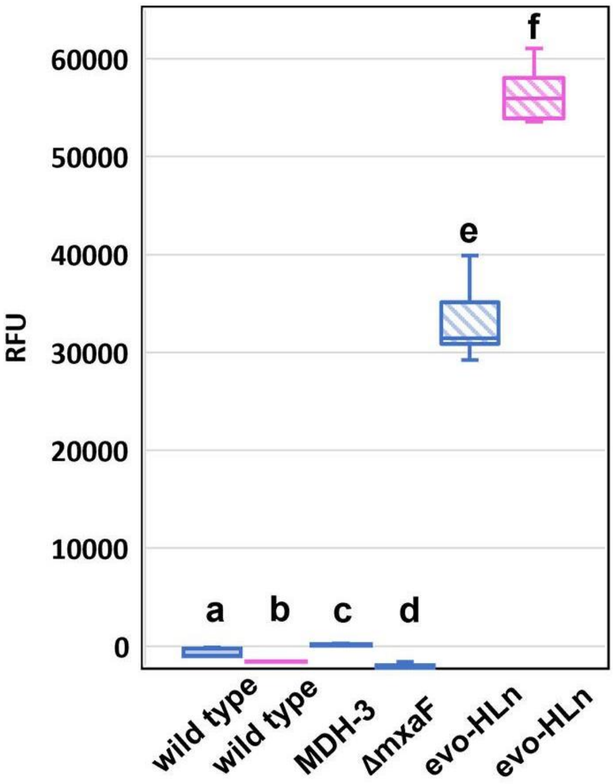

Supplementary Figure 3. Response of *exaF* promoter in *evo*-HLn with La and Gd. *evo*-HLn exhibits higher *exaF* promoter activities with both light and heavy Ln. Wild type, MDH-3, Δ*mxaF* and *evo*-HLn carrying a *exaF* promoter-*yfp* reporter fusion construct were grown with methanol and either no Ln (black), La (blue), or Gd (pink) to an OD of ~1.0 at 600 nm and promoter readout was measured as fluorescence. Box and whisker plot shows the interquartile range of RFU determined for 3 biological replicates with 2 technical replicates of each fluorescence measurement. Whiskers show the minimum and maximum values. For each strain and growth condition, readout from the promoter-less construct was subtracted as background fluorescence.

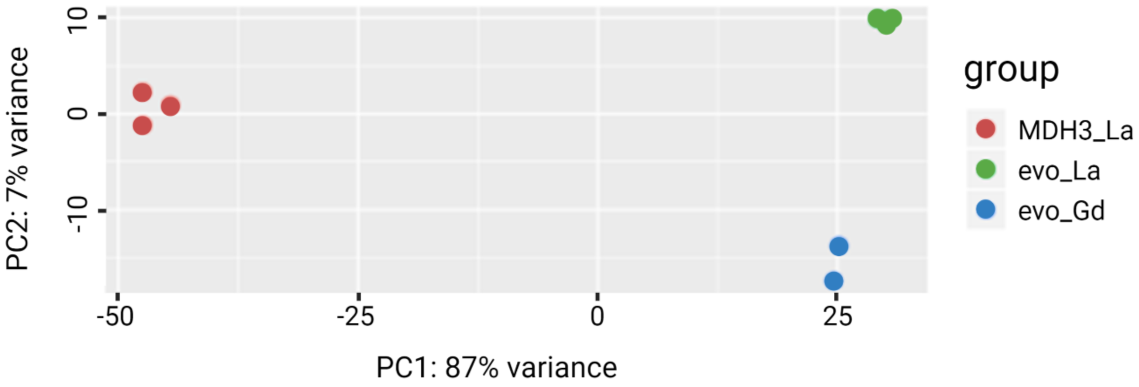

Supplementary Figure 4. Principal component analysis of log2-transformed expression data for all coding sequences used for differential gene expression analysis. MDH-3_La, MDH-3 grown with µM $LaCl_3$; evo_La, *evo*-HLn grown with µM $LaCl_3$; evo_Gd, *evo*-HLn grown with 2 µM $GdCl_3$. All cultures were grown with 50 mM methanol in PIPES minimal medium. Not all data points are completely visible. Analysis was performed using DESeq2.

REFERENCES

Marx, Christopher J., Brooke N. O'Brien, Jennifer Breezee, and Mary E. Lidstrom. 2003. "Novel Methylotrophy Genes of *Methylobacterium extorquens* AM1 Identified by Using Transposon Mutagenesis Including a Putative Dihydromethanopterin Reductase." *Journal of Bacteriology* 185 (2): 669–73. https://doi.org/10.1128/jb.185.2.669-673.2003.

Nunn, D. N., and M. E. Lidstrom. 1986. "Isolation and Complementation Analysis of 10 Methanol Oxidation Mutant Classes and Identification of the Methanol Dehydrogenase Structural Gene of *Methylobacterium* Sp. Strain AM1." *Journal of Bacteriology* 166 (2): 581–90. https://doi.org/10.1128/jb.166.2.581-590.1986.

Puri, Aaron W., Sarah Owen, Frances Chu, Ted Chavkin, David A. C. Beck, Marina G. Kalyuzhnaya, and Mary E. Lidstrom. 2015. "Genetic Tools for the Industrially Promising Methanotroph *Methylomicrobium Buryatense*." *Applied and Environmental Microbiology* 81 (5): 1775–81. https://doi.org/10.1128/AEM.03795-14.

Skovran, Elizabeth, Alexander D. Palmer, Austin M. Rountree, Nathan M. Good, and Mary E. Lidstrom. 2011. "XoxF Is Required for Expression of Methanol Dehydrogenase in *Methylobacterium extorquens* AM1." *Journal of Bacteriology* 193 (21): 6032–38. https://doi.org/10.1128/JB.05367-11.

Skovran, Elizabeth, Charumathi Raghuraman, and Norma Cecilia Martinez-Gomez. 2019. "Lanthanides in Methylotrophy." *Current Issues in Molecular Biology* 33 (June): 101–16. https://doi.org/10.21775/cimb.033.101.

Vu, Huong N., Gabriel A. Subuyuj, Srividhya Vijayakumar, Nathan M. Good, N. Cecilia Martinez-Gomez, and Elizabeth Skovran. 2016. "Lanthanide-Dependent Regulation of Methanol Oxidation Systems in *Methylobacterium extorquens* AM1 and Their Contribution to Methanol Growth." *Journal of Bacteriology* 198 (8): 1250–59. https://doi.org/10.1128/JB.00937-15.
